# Multiple *var2csa*-Type PfEMP1 Genes Located at Different Chromosomal Loci Occur in Many *Plasmodium falciparum* Isolates

**DOI:** 10.1371/journal.pone.0006667

**Published:** 2009-08-19

**Authors:** Adam F. Sander, Ali Salanti, Thomas Lavstsen, Morten A. Nielsen, Pamela Magistrado, John Lusingu, Nicaise Tuikue Ndam, David E. Arnot

**Affiliations:** 1 Centre for Medical Parasitology, Department of International Health, Immunology & Microbiology, Faculty of Health Sciences, University of Copenhagen & Department of Infectious Diseases, Copenhagen University Hospital (Rigshospitalet), Copenhagen, Denmark; 2 JMP-ENRICA Project, National Institute for Medical Research, Korogwe Laboratory, Tanga, Tanzania; 3 Institut de Recherche pour le Developpment, UR010, Universite Paris Descartes, Paris, France; 4 Institute of Immunology & Infection Research, School of Biology, University of Edinburgh, Edinburgh, Scotland, United Kingdom; Sabin Vaccine Institute, United States of America

## Abstract

**Background:**

The *var2csa* gene encodes a *Plasmodium falciparum* adhesion receptor which binds chondroitin sulfate A (CSA). This *var* gene is more conserved than other PfEMP1/*var* genes and is found in all *P. falciparum* isolates. In isolates 3D7, FCR3/It4 and HB3, *var2csa* is transcribed from a sub-telomeric position on the left arm of chromosome 12, but it is not known if this location is conserved in all parasites. Genome sequencing indicates that the *var2csa* gene is duplicated in HB3, but whether this is true in natural populations is uncertain.

**Methodology/Principal Findings:**

To assess global variation in the VAR2CSA protein, sequence variation in the DBL2X region of *var2csa* genes in 54 *P.falciparum* samples was analyzed. Chromosome mapping of *var2csa* loci was carried out and a quantitative PCR assay was developed to estimate the number of *var2csa* genes in *P.falciparum* isolates from the placenta of pregnant women and from the peripheral circulation of other malaria patients. Sequence analysis, gene mapping and copy number quantitation in *P.falciparum* isolates indicate that there are at least two loci and that both *var2csa*-like genes can be transcribed. All VAR2CSA DBL2X domains fall into one of two distinct phylogenetic groups possessing one or the other variant of a large (∼26 amino acid) dimorphic motif, but whether either motif variant is linked to a specific locus is not known.

**Conclusions/Significance:**

Two or more related but distinct *var2csa*-type PfEMP1/*var* genes exist in many *P. falciparum* isolates. One gene is on chromosome 12 but additional *var2csa*-type genes are on different chromosomes in different isolates. Multiplicity of *var2csa* genes appears more common in infected placentae than in samples from non-pregnant donors indicating a possible advantage of this genotype in pregnancy associated malaria.

## Introduction

The *var2csa* gene is the best characterized of the PfEMP1/*var* genes and the protein it encodes is a parasite receptor for binding to human placental CSA and thought to play an important role in the pathogenesis of pregnancy associated malaria (PAM) [1]–[3]. VAR2CSA proteins are large (∼350 kDa), antigens, exposed to host antibodies on the erythrocyte surface membrane [4], [5]. They are more variable than most viral antigens known to bind endothelial receptors, but unusually conserved compared to other members of the extremely diverse PfEMP1 antigen family. The *var2csa* genes are relatively distantly related to other *var* genes and constitute a distinct group whose recombination with other *var* genes is suppressed [6], [7]. The *var2csa* gene is also one of only two *var* genes (the other being *var1csa*) which are found in all three of the original *P.falciparum* reference genome sequences [7]. The *var2csa* gene is the only PfEMP1/*var* gene sufficiently conserved to have a recognizable homologue in *P.reichenowi*, the chimpanzee *Plasmodium* species that is *P. falciparum's* closest evolutionary relative [8].

Because it is essential to adhesion of malaria parasites to the placenta [9], VAR2CSA has been proposed as the antigen in an adhesion-blocking vaccine to protect women against malaria during pregnancy [10]–[12]. There is therefore interest in defining the domains involved in adhesion to CSA, such as the DBL2X region [13]. To define variability in the VAR2CSA DBL2X domains we analyzed these sequences in *var2csa* genes in *P.falciparum* samples taken from placentae at delivery and from the peripheral circulation of malaria patients, combining these with sequences from database sources.

DBL2X polymorphism consists of defined blocks of variability, divided by regions of sequence conservation, a pattern of variation generally observed in PfEMP1 protein domains [14]–[16]. However the sequence of one substantial region of the VAR2CSA DBL2X domain exists in two dimorphic types. One explanation for this striking dimorphic sequence motif (DSM) might be that there is more than one *var2csa*-type gene per haploid genome, a tentative conclusion also reached based on the *var* gene sequence assembly from the HB3 isolate *P.falciparum* genome sequencing project [7]. This ‘*multiple var2csa genes*’ hypothesis was therefore tested by mapping the position of *var2csa*-type genes in pulsed field gel chromosome separations of different parasite isolates. Following confirmation of the existence of two separate loci, a quantitative real-time PCR assay, based on the 2^−ΔΔ^ Ct method, was developed to count the numbers of *var2csa*-type genes in *P.falciparum* clinical isolates.

## Materials and Methods

### Origin and maintenance of *P.falciparum* isolates

Six placental field-isolates (Tz745, Tz748, Tz752, Tz755, Tz788 and Tz796) and five long-term cultured clones (HB3, FCR3/It4, DD2, 7G8 and 3D7) were grown. Modified Trager-Jensen medium consisting of blood group O^+^ red blood cells (5% haematocrit), supplemented with 25 mM sodium bicarbonate, 0.125 g L^−1^ gentamycin and 0.125 g L^−1^ Albumax II was used. Flasks were kept at 37°C and gassed with 2% oxygen, 5% carbon dioxide in nitrogen. Parasites were harvested at around 5% parasitaemia. Cultures were tested for isolate integrity using nested GLURP and MSP-2–specific primers to measure clone multiplicity by PCR. DBL2X sequences were from placental blood samples collected at delivery in Guediawaye maternity ward, Senegal [17] and stored on filter paper, or extracted from clinical samples collected in Daraweesh, Sudan [18] or Korogwe, Tanzania [19]. Other sequences are from PlasmoDB.

### PCR amplification and sequencing of *P.falciparum* genomic DNA

Genomic DNA was extracted from placental blood samples on filter paper using the Chelex method [20] or extracted directly from patient blood. The *var2csa* gene was amplified using various combinations of domain specific primers (Table 1). Multi-sequence alignments were made using MAFFT software (http://align.bmr.kyushu-u.ac.jp/mafft/software/) with the “G-INS-I” setting for global alignments, corrected manually.

**Table 1:** PCR amplification primers.

Gene:	Primer ID/application:	Primer sequence:	Tm:
var2csa-	557 (Sequencing)	5′ CGGAATTCAAATGCGACAAATGTAAAT	54
(DBL2X)	558	3′ ATTTGCGGCCGCCTGATTGTACACATTTATT	60
	569	5′ CGGAATTCGGATCTAGTTCTAATGGTAGTTGT	61
	570	3′ ATTTGCGGTCGCAATGTTTGAAAAACGAATC	59
	653	5′ CGGAATTCCTTCAAGAAAATTGTAGTG	55
	654	3′ ATTTGCGGCCGCGTGTGGTCAATCCCTATAT	64
var2csa-	A763 (Sequencing)	5′ GAAATTGACAATGCAATAA	56
(DBL5e)	A764	3′ CTTCAAGTTCAGCTGGAAT	54
Tz748-var2csa-	748A (Transcript quant. assay)	5′ ATATTAAAAGATGTAAAGGAACCG	49
(DBL5e)	748A	3′ TTTCTTTTTCGTTGTCTTCATTG	48
	748B	5′ AATGTATTTAACAATGCAAATGA	47
	748B	3′ CTTCATTTCCGATGTTTGTATAT	48
Tz745-var2csa-	745A (Transcript quant. assay)	5′ AACAAAACTTGGAGGCAAATG	49
(DBL5ε)	745A	3′ GAGATCCAGCAGTACCAC	50
	745B	5′ AGG AAT GTG GAA ACA AAT GTA	47
	745B	3′ GGATCCGGCAGTACCAGT	53
seryl-tRNA synthetase	P90 (Transcript quant. Assay)	5′ AAGTAGCAGGTCATCGTGGTT	52
(PF07_0073)	P90	3′ TTCGGCACATTCTTCCATAA	48
var2csa-	T12 (PCR copy number assay)	5′ AATGGGACAAACAAAAAACAAAATAT	51
(DBL3x)	T13	3′ GCTGATATACATTCAGGATAATTTTC	52
aldolase	P61 (PCR copy number assay)	5′ TGT ACC ACC AGC CTT ACC AG	54
(PF14_0425)	P61	3′ TTCCTTGCCATGTGTTCAAT	48

### Phylogenetic analysis of the domain variants

Phylogenetic analyses were conducted using MEGA version 4.0.2. Input data were sequence alignments of VAR2CSA DBL2X and DBL5ε amino acid sequences. Phylogenetic trees were constructed both as unrooted neighbor joining (NJ) and maximum parsimony trees using a Dayhoff matrix substitution model. Inferred phylogeny was validated by bootstrapping (1,000 replications).

### Pulsed field gel electrophoresis and Southern blotting

*P.falciparum* chromosomal DNA blocks were prepared as described [21]. Pulsed field gel electrophoresis was carried out using a Bio-Rad™ CHEF DRII apparatus (0.5X TBE buffer, circulated at 14°C). Chromosomes were separated on 0.7% agarose gels, initially ramping the pulse interval from 90–300s for 24 hours at 4.2 V/cm, followed by 300–720 s for 43 hours at 3.0 V/cm. For Southern blotting, chromosomes were hydrolyzed in the gel (0.25N HCl for 30 mins), prior to denaturation, renaturation and 10X SSC transfer onto Hybond N^+^ nylon membranes (Amersham GE Healthcare). The DBL3X probe was labeled with dioxygenin-dUTP by PCR using primers forward (pos.4498–4525) and reverse (pos. 4613–4642), based on the 3D7 *var2csa* sequence [22]. Membranes were pre-hybridized overnight at 42°C in 5X SSC, 5X Denhardt's, 0.5% SDS, 20 mM Tris-HCl pH 7.5, 10% Dextran Sulphate, 35% formamide. Hybridization was carried out in the same buffer with dioxygenin (DIG) (Roche) labeled probe, at 42°C overnight. The blot was washed in SSC at 60°C, with increasing stringency washes, ending with 0.1 X SSC, 0.1% SDS, at room temperature.

### Quantitative Real-Time PCR (Q-RT PCR)

Q-RT-PCR was performed using the Rotorgene 6000, version 1.7 system (Corbett Research). Reactions were prepared in 20 µL using Quantitec SYBER Green PCR master mix (Qiagen) and primer concentrations of 1 µM. PCR cycling was 95°C for 15 mins, followed by 40 cycles of 95°C for 30 s, 54°C for 20 s and 65°C for 40 s, with final extension at 68°C for 40 s. The cycle threshold, the PCR cycle at which the product-derived fluorescence intensity crosses a threshold value, was set at 0.025, which reflected the optimal PCR parameters across all runs.

### Selection and validation of primers for 2^−ΔΔ^ Ct analysis

The *var2csa* and *aldolase* primers (see Table 1) were selected to target known conserved sequences [23]. Primers were optimised to avoid self annealing and hairpin loops formation. Oligonucleotide lengths and melting temperatures were equalized as much as possible and specificity of amplification measured by melting-curve analysis. Primers were BLAST searched against *P.falciparum* and human databases to ensure specificity. All PCR reactions were checked for the amplification of a single band of the expected size.

### The 2^−ΔΔ^ Ct method of relative gene copy number estimation

The 2^−ΔΔ^ Ct method of relative quantitation [24] was adapted to estimate copy number of the *var2csa* gene. For this calculation to be valid, the amplification efficiency (E) must be close to 100% and E values for target and reference genes (the relative efficiency) must be approximately equal. The measurement of copy number of a target gene in an unknown sample requires a ‘calibrator’ genome with known copy number of both target and reference genes. FCR3/It4 was used as the calibrating parasite genome and the *var2csa* copy number was determined relative to FCR3/It4, using equations (1) 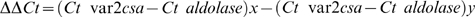, where *x*  =  unknown sample and *y*  =  *P.falciparum* FCR3/It4, and (2) in which the copy number of the unknown target gene is expressed as 2^−ΔΔ^ Ct.

The assay is particularly sensitive to the quality of template DNA preparations. The intrinsic variation between different DNA samples was assessed by making ten separate extractions of DNA, both from filter paper and infected blood, of the FCR3/It4 calibrator, and measuring their Ct values, in triplicate, in three separate runs. The total error, including variation between triplicates, DNA preparations and runs is then included in subsequent calculations of target gene copy numbers, by computing the upper and lower limits of the 95% confidence intervals of both the unknown sample and calibrator. Upper and lower 95% limits of the ΔΔ Ct value were then used to give a more accurate 95% confidence limit on the copy number estimate, given as [2^∧−ΔΔCt High^− 2^∧−ΔΔCt Low^]. Three conservative sample inclusion criteria were used. Therefore samples were excluded if the Ct value was >26, if triplicates had a CV >1% and if samples had a 95% confidence copy number interval between integer values 1 and 2.

### Transcript quantitation assay

To test whether either or both of the *var2csa*-type genes were expressed as mRNA in two locus isolates, variant specific primer to amplify reverse transcribed cDNA were designed (Table 1). To compare the transcription of each gene, the amplification efficiency of the two primer pairs were first determined by real-time measurements of serial 10 fold dilutions of plasmid DNA carrying inserts of the two *var2csa*-type genes (Tz745 and Tz748). Quantitative measurements were then made by comparing the Ct values of the two *var2csa*-type genes in each isolate with that of the endogenous housekeeping gene (*seryl-tRNA synthetase*, PF07_0073).

## Results

### Variation and conservation in *var2csa*-type genes and proteins

**Figure 1:**
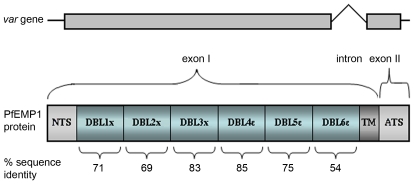
Structure of the *var2csa* gene and protein. The gene and its exon structures are shown above a protein diagram which has been subdivided into predicted domains [71]. The approximate amount of variation (expressed as percentage amino acid identity) in these domains is shown below each domain block. The sequence similarities were calculated based on the genome sequencing data available (from 3D7, FCR3/It4, HB3, Dd2, PfClin, and *P.reichenowi*). The trans-membrane region and conserved (>95%) intracellular acidic terminal sequence (ATS) are not included in the analysis.

Figure 1 shows a diagram of the VAR2CSA gene and protein. Salanti *et al* [25] noted that this gene differs from the other *var* genes in that some of its domains do not conform to the sequence motif spacing-based classification schemes for CIDR and DBL domains used in other PfEMP1 proteins [26]. An estimate of VAR2CSA domain conservation between parasite isolates, calculated on the basis of amino acid sequences, is given by the percentage sequence identity figure shown below each domain. These similarity estimates are based on nine complete *var2csa* genes assembled in the PlasmoDB database [27] and the *P.falciparum* genome sequencing projects at the Sanger and Broad Institutes. Most PfEMP1/*var* genes have <50% amino acid sequence identity between individual DBL domains [7], but significantly higher levels of conservation between isolates are seen in all VAR2CSA domains. The most conserved regions are the central DBL3X and DBL4ε domains. The N and C-terminal regions are somewhat more variable, the membrane-proximal DBL6ε being the most polymorphic domain. Domain functions remain unclear although DBL5ε binds IgM [28]–[30] but whether binding to CSA is mediated by a single or multiple domains is controversial [31]–[36]. Host antibodies bind antigenic structures throughout the molecule [37], [38] and antibodies specific for DBL4ε have been shown to inhibit ligand binding [39].

### Variation in the DBL2X domain of VAR2CSA

**Figure 2:**
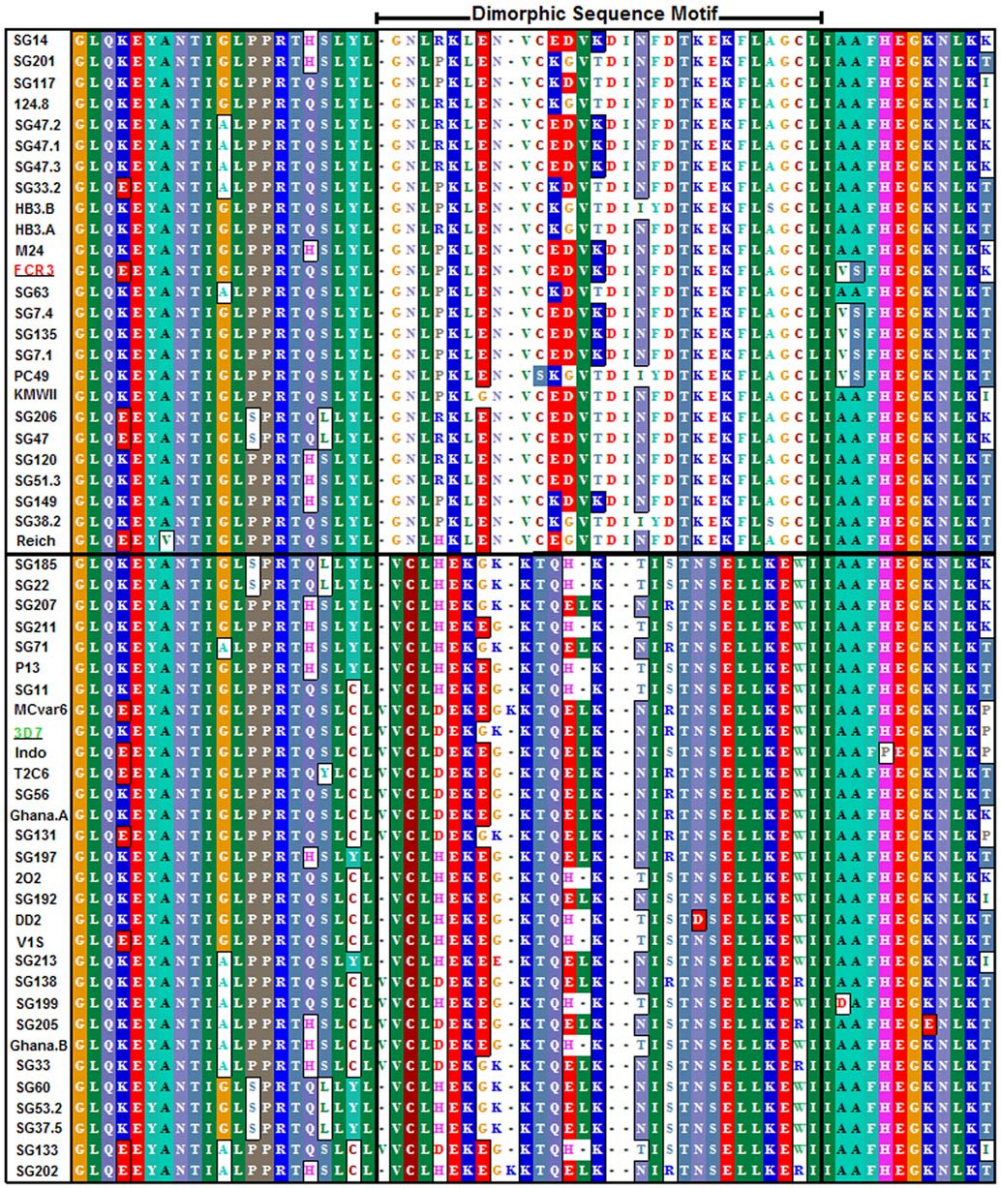
Dimorphic sequence motif in the DBL 2X domain of VAR2CSA. The multiple sequence alignment covers a region of 60–62 amino acids and includes 37 VAR2CSA DBL2X sequences from Senegalese (SG) placental isolates (accession numbers GQ358101 – GQ358135) and 18 database-derived sequences, including *P.reichenowi* (*Reich*). This region includes amino acids 580–641 of the VAR2CSA sequence encoded by the 3D7 PFL0030c gene. Color blocks mark positions of the alignment with >55% amino acid sequence identity. The dimorphic sequence motif (DSM) is shown between positions 50–78. All sequences contain one or the other DSM variant. A solid line separates FCR3/It4-type sequences from 3D7 type sequences.

As part of a study of variation in VAR2CSA, polymorphism in the VAR2CSA DBL2X domain was analyzed. The alignment of the DBL2X domain shows the pattern of interspersed conserved and variable sequences previously seen in the VAR2CSA DBL3X domain [40]. The full alignment of 55 different ∼340 amino acid DBL2X sequences is shown in Figure S1. However it is striking that a relatively large region of the DBL2X domain has one or the other of two variants of a conserved sequence. This clearly differentiates these sequences into two distinct groups. Figure 2 details a ∼62 a.a comparison between 55 VAR2CSA DBL2X domain sequences highlights this dimorphic sequence motif (DSM). Color blocks show regions of >55% sequence identity. Interestingly, the *P.reichenowi* VAR2CSA DBL2X has conserved this motif and has the FCR3/It4-type variant of the dimorphism. The two closely related sequences derived from the HB3 genome sequencing project are labeled HB3.A and HB3.B. Both are of the FCR3/It4 DSM sub-type.

### Structural models of the VAR2CSA DBL 2X domain

**Figure 3:**
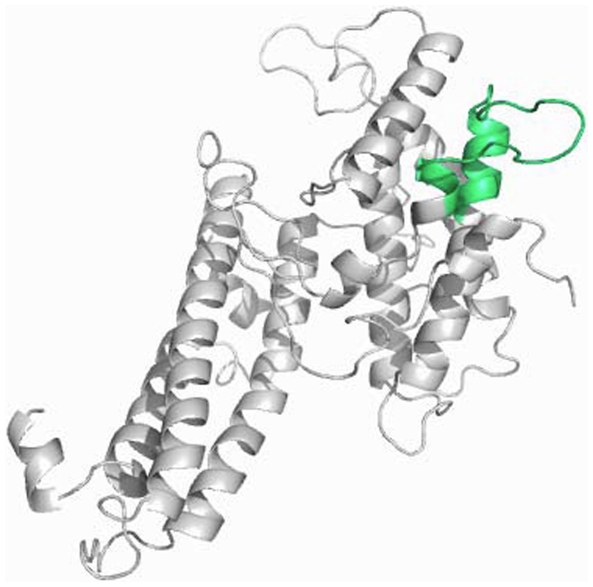
Three-dimensional model of the VAR2CSA DBL2X domain. The three dimensional model of the DBL2X domain displays the position of the dimorphic sequence motif (DSM). The model was constructed using the F1 domain of the *P.falciparum* Erythrocyte Binding Antigen (EBA 175, Protein data bank code 1ZRO) as a structure template. The VAR2CSA amino acid sequence used is the 3D7 genome sequence (PFL0030c amino acids 535–878).

Structural models have been made of the DBL2X and DBL3X domains [41], [42], based on similarity to the DBL domain of the *P.falciparum* EBA 175 antigen [43]. Recent crystal structures of VAR2CSA DBL3X [44], [45] indicate that the lower-resolution, homology-based model nonetheless agrees well with the experimentally determined structure. Figure 3 illustrates a 3-D model of the DBL2X domain (which has not yet been analyzed by crystallography), similar to that recently published by Bockhorst *et al* [46], highlighting the position of the DSM which covers an exposed loop and part of an α-helical region of this domain.

### Phylogenetic relationships between VAR2CSA DBL2X domains

**Figure 4:**
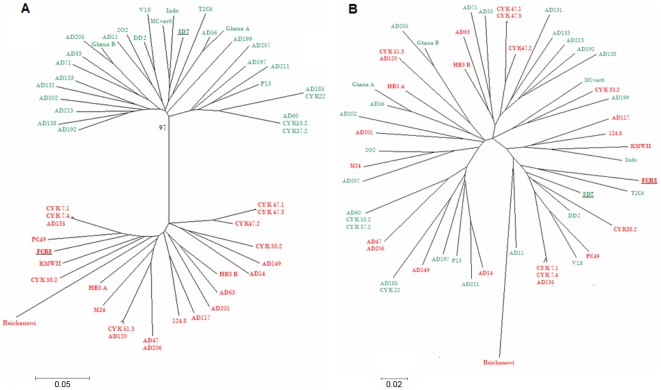
Phylogenetic relationships between 44 VAR2CSA DBL2X sequences. A. Neighbor joining tree illustrating the phylogenetic relationship between the sequences, with representatives of each of the two DSM variants marked in separate colors. 3D7-type sequences are shown in red and FCR3/It4-type in green. The bootstrap value is shown at the main bifurcation. B. Neighbor joining tree based on the same sequences, after excision of the DSM region. The 3D7/red, FCR3/It4/green variant-type coding is maintained.

The dimorphism of DBL2X domains was investigated by a standard type of phylogenetic analysis based on the assumption that sequences are evolving largely by mutation. This has been cogently criticized as underestimating the role of very high levels of recombination in generating hyper-variable sequence blocks in *P.falciparum* PfEMP1/*var* genes [47]. However, by the standards of PfEMP1/*var* genes, the VAR2CSA DBL2X domain is not a hypervariable region and in fact shows some highly conserved features, as discussed below. Figure 4 shows the constructed neighbor-joining trees, based on 54 *P.falciparum* and one *P.reichenowi var2csa* DBL2X sequences. Figure 4A confirms that these DBL2X sequences will cluster into two distinct phylogenetic subgroups (bootstrap value 97). Sequences fall into one of two reference groups, the 3D7 DSM type, shown in green, or the FCR3/It4 DSM type, shown in red. To test whether this is due to the DSM, or other less obvious variation, the sequences were re-tested, after excising the DSM sequence from the alignments. The neighbor joining tree shows that the division of the sequences into two groups disappears and the branching of the tree is similar to the relationship observed between variants of other DBL domains [48]. Unsurprisingly, the branch lengths support a more distant relationship between the *P.reichenowi* sequence and all *P.falciparum* sequences but the conservation of the DSM still results in this particular *P.reichenowi* sequence clustering with FCR3/It4 sub-type sequences.

The majority of the sequences in this analysis are from Dakar, Senegal. However, Figure 4 shows that the branching pattern also accommodates and separates the other African isolates used (*e.g.* Ghana A and B and SD2O2 and SD124.8 from Sudan), the database sequences (DD2, MC, FCR3/It4, HB3 and 3D7) and the *P. reichenowi* isolate. These isolates originate from other areas of Africa, Central America and S.E. Asia and indicate that a dimorphic sequence influenced phylogeny is a general feature of the DBL2X domain of *var2csa*-type genes.

### Mapping the *var2csa* gene onto pulsed field gel separated chromosomes

Alternative hypotheses could explain the existence of the two VAR2CSA DBL2X domain types. Either a single *var2cs*a gene locus encodes a dimorphic series of allelic variants, or multiple loci encode related *var2csa*-type genes, each having a particular variant of the DSM. The *var2csa* gene has been mapped to the sub-telomeric region of the left arm of chromosome 12 in isolates FCR3/It4, 3D7 and HB3 but is not known if this location is universal. Genome sequencing projects have detected, but not mapped, two *var2csa*-type genes in the HB3 isolate [7]. To resolve these uncertainties, a conserved portion of the *var2csa* gene was used to map the location of the *var2csa* locus in Southern blots of pulsed field gel (PFG) separated *P.falciparum* chromosomes.

**Figure 5:**
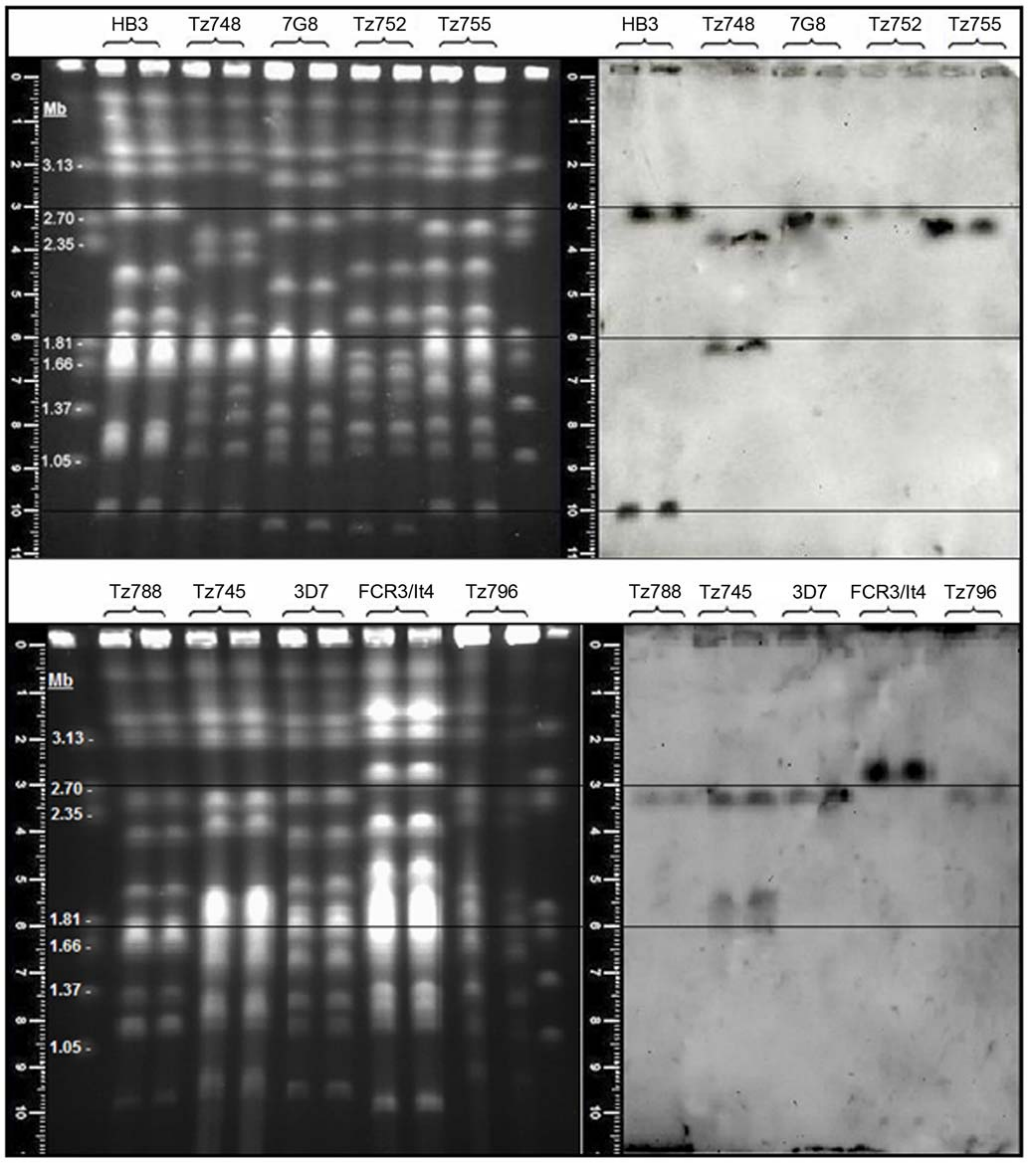
Chromosomal location of *var2csa*-type loci in *P.falciparum* isolates. Conditions for the separation of the chromosomes and DIG labeling and detection of the *var2csa* DBL3X domain-derived probe sequence are given in the Materials and Methods. Gel measuring rulers and superimposed alignment lines are shown. Marker chromosomes are from *Hansenula wingei*. Note that the two uppermost faint bands are not chromosomes but a PFG artifact. HB3, 3D7, FCR3/It4 and 7G8 are long term laboratory isolates whilst Tz745, Tz748, Tz752, Tz755, Tz788 and Tz796 are recent field isolates from Korogwe, Tanzania.

Figure 5 illustrates a separation of the 14 *P.falciparum* chromosomes and the corresponding Southern blot, probed with a DBL3X-derived *var2csa*-specific gene probe. To compensate for gel distortions each sample was run in duplicate. The PFG illustrates the chromosome length polymorphisms that are a feature of *P.falciparum* [49]. Confirming earlier experiments, chromosome 12 contains a *var2csa* gene locus, hybridized by the *var2csa* gene probe in all 10 isolates shown in Figure 5. Two of the isolates, HB3 and Tz748, have one additional locus hybridizing the *var2csa* probe, on chromosome 1 in HB3 and on what is probably chromosome 8 in Tz748 (hybridizing to the upper region of the poorly resolved chromosomes 5–9, Fig. 5A). The Tz745 has a distinct chromosome 12 *var2csa* locus and at least one and quite possibly two additional hybridizing loci in the poorly resolved chromosome 5–9 region (Fig. 5B).

Gene mapping thus shows that both long-term laboratory isolate HB3 and recent field isolates such as Tz745 and Tz748 have additional loci containing a second *var2csa*-type gene. We think it likely that isolates containing three *var2csa*-type genes also exist (Fig. 5B, see Tz745). Since the presence of more than one clone in the culture can be easily detected by PFG analysis, these results also indicate that detection of two *var2csa*-type genes in these isolates is not caused by the presence of multiple clones in the original isolate.

Genome sequences exist for four established laboratory clones of *P.falciparum* (3D7, FCR37It4, HB3 and Dd2), for one clinical isolate of Ghanaian origin, and for *P.reichenowi* [50]–[52]. In HB3, two complete *var2csa* genes have been assembled (http://www.broad.mit.edu). This agrees with our gene mapping data for HB3 although at this stage of annotation, the two *var2csa* loci have been placed in head to head orientation on chromosome 12. Our data indicate that the second HB3 *var2csa* gene is in fact on chromosome 1. Hybridization signals should be proportional to target copy number and the relative intensities observed do not indicate the presence of multiple *var2csa*-type genes on chromosome 12 in HB3.

### PCR assay estimating *var2csa* gene multiplicity in *P.falciparum* isolates

PFG-based gene mapping is an unambiguous but unwieldy tool for estimating the number of *var2csa*-type genes in large numbers of clinical isolates because these are difficult to establish and maintain in culture. It is therefore desirable to have an assay that can be used to measure gene copy number in DNA isolated from preserved blood. The 2^−ΔΔ^ Ct assay does this by normalizing the amount of a variable-copy target gene, against an endogenous single copy gene. After calibration, target gene number in samples can be expressed as *n*-fold changes in target gene copies, relative to the copy number of the target gene in an endogenous ‘spike’ (known to be 1). Based on our gene mapping and the genome project analysis, *P.falciparum* FCR3/It4 has the requisite single copy *var2csa* gene.

**Figure 6:**
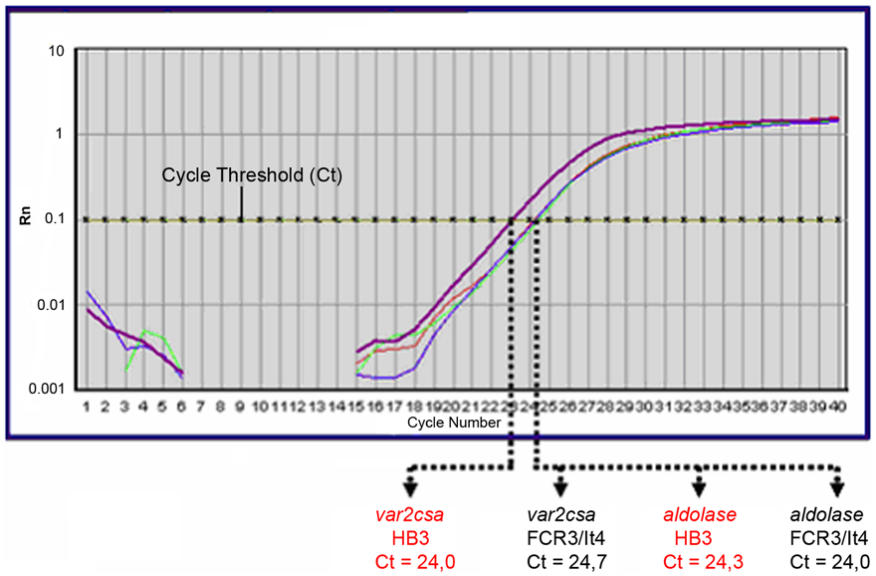
2^−ΔΔ^ Ct assay for counting *var2csa*-type gene copy number. Cycling and assay conditions are detailed in the Materials and Methods section. The cycle thresholds (Ct) for detection of *var2csa*-type target genes and *aldolase* reference genes are shown as the points where the signal crosses an experimentally predetermined threshold value, in a graph of Rn (normalized reporter by fluorescence) against the number of PCR cycles.

Figure 6 shows a 2^−ΔΔ^ Ct assay graph of DNA quantity dependent fluorescence (Rn) versus PCR cycle number. Amplification of the *var2csa* gene crosses the pre-determined cycle threshold at Ct = 24.0 for *P.falciparum* HB3. This occurs at Ct = 24.7 for *P.falciparum* FCR3/It4. The corresponding values for the *aldolase* gene are Ct 24.3 and 24.0 respectively. Using the ΔΔ Ct equations;
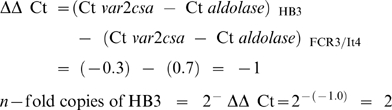



For clone HB3, the 2^−ΔΔ^ Ct assay estimated *var2csa* copy number agrees with the gene mapping and the genome assembly. With other isolates known to have two loci (Tz748 and Tz745), the assay agrees with the PFG gene mapping. Calibrated with a reference genome ‘spike’, and including primers for a single copy housekeeping gene for normalization, the 2^− ΔΔ^ Ct assay thus appears valid as a gene counting method for *var2csa* genes.

The polyclonal nature of many *P.falciparum* infections [53] complicates gene counting and makes sequencing an unreliable method for estimating gene copy numbers. The PCR assay however can not over-estimate the number of isolates containing clones with multiple *var2csa*-type genes, since a mixture of single and multi-copy isolates can only bring the copy number estimate closer to one. Nevertheless, polyclonality precludes the calculation of actual target gene numbers in mixed genotype infections. Instead, the 2^-ΔΔ^ Ct allows estimation of the proportion of isolates containing parasites which have multiple *var2csa*-type genes/haploid genome.

We have analyzed a sample set consisting of 43 blood samples from children in Korogwe, Tanzania, 36 placental blood samples from Korogwe and 32 peripheral blood samples from non-pregnant malaria cases in Daraweesh, Sudan. The 2^−ΔΔ^ Ct assay results, showing the proportion of samples containing parasites with multiple *var2csa*-type genes are presented in Table 2. Parasites derived from the placentae were more likely to contain clones which have multiple *var2csa*-type genes, although this inequality was not statistically significant. The existence of an advantage of the *var2csa* multi-locus genotype in the malaria in pregnancy syndrome is therefore neither established nor excluded.

**Table 2:** *var2csa* gene multiplicity in samples from placentae at delivery and from non-pregnant *P.falciparum* donor samples.

Sample origin	Number of samples	Placental/Non-pregnant samples	Proportion of samples with multiple *var2csa*-type gene per genome
Korogwe, Tanzania	36	Placental	31%, (11/36)
Korogwe, Tanzania	43	Non-pregnant	14%, (6/43)
Daraweesh, Sudan	32	Non-pregnant	15%, (5/32)
**Total**	111	all	20%, (22/111)

### The relationship between *var2csa*-type genes located in the same genome

To discover if the multiple *var2csa*-type genes found in some *P.falciparum* genomes represent recently duplicated paralogs, we sought to analyze whether genes present in the same genome are more closely related to each other than they are to other *var2csa*-type genes. Such an analysis requires pre-identification of monoclonal isolates with multiple *var2csa* loci. Assessing the degree of relationship between *var2csa*-type genes also requires analyzing domain of the protein that show no evidence of dimorphism, since isolates with two loci, each with the same dimorphic region (*e.g.* HB3) are less easily recognized as distinct loci. DBL5ε domain sequences were therefore used for this analysis of the relationship between *var2csa*-type genes in the same genome. After identifying eight monoclonal samples with multiple *var2csa*-type genes, their DBL5ε coding regions were amplified, sub-cloned and sequenced. These were compared with 25 *var2csa* DBL5ε-type sequences to estimate the relatedness of *var2csa*-type genes known to exist within the same genome, as compared to other *var2csa*-type genes. None of the *var2csa*-type sequences from multi-copy genomes had any unique polymorphisms absent from other DBL5ε sequences (data not shown).

**Figure 7:**
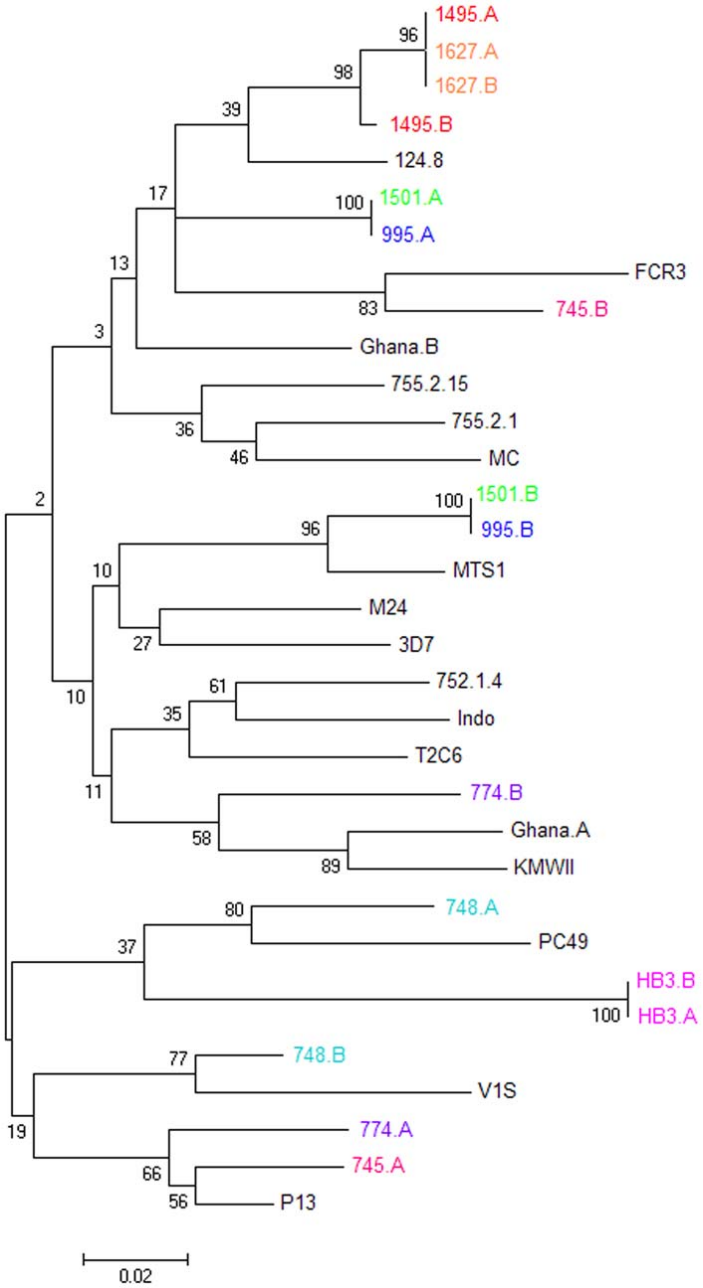
Phylogenetic relationships between VAR2CSA DBL5ε sequences. A neighbor joining tree showing the relationships between 33 different VAR2CSA DBL5ε sequences, including eight sequences derived from samples which are known to have two or more *var2csa*-type genes/haploid genome. Sequences derived from the same genome are presented in the same color. Bootstrap values for each clade are shown at the nodes of the tree.

The phylogenetic relationship between DBL5ε sequences derived from the same genome and an essentially random sample of other DBL5ε sequences is shown in Figure 7. The tree does not support the hypothesis that multiple *var2csa*–type genes are the result of recent duplication events in most isolates, although the HB3 and 1627 sequences are quite closely related and could represent relatively more recent duplications. In at least some of the genomes analyzed, the two genes appear to have been evolving independently for sufficient time to have diverged considerably.

### Are both *var2csa* genes capable of being transcribed?

To test whether more than one of the *var2csa*-type genes are being transcribed into mRNA in multi-loci isolates such as Tz745 and Tz748, *var2csa* variant-specific primers were designed to amplify cDNA from these cultures. Both parasite isolates are monoclonal, as judged by PCR genotyping and pulsed field gel electrophoresis and both bind CSA on BeWo syncytiotrophoblastic cells. BeWo adhesion-selected Tz745 and Tz748 also show the characteristic parity-dependent serological recognition phenomenon [54]. The amplification efficiency of variant specific primers was determined. cDNA of the *var2csa*-type genes from both isolates was then amplified using each primer pair, and assayed relative to the amplification of *seryl-tRNA synthetase*. The results showed that in both adhesion-selected cultures, there is specific transcription from each of two *var2csa-*type genes (Ct _745.A_ = 16.1; Ct _745.B_ = 12.3), (Ct _748.A_ = 17.1; Ct _748.B_ = 16.1) and the transcript levels of both genes are increased relative to those measured for the housekeeping gene (Ct _745_ = 18.0; Ct _748_ = 17.7), after adhesion selection. Each gene thus appears to be transcription-competent and, in these isolates is neither is a pseudo gene.

## Discussion

Sequencing projects have been reported for six *P.falciparum* genomes, each complete and annotated to a different degree. There is generally incomplete coverage of the repeat-rich telomeric sequences (where many *var* genes lie) and annotation of the multi-gene families such as PfEMP1 and the RIFINS has proven difficult and time-consuming. The first report that the HB3 genome sequencing project detected two *var2csa*-type genes indicated that these genes are very similar and closely linked on chromosome 12 and thus potentially recent duplications. The biological relevance of such events is uncertain although a recent study using monoclonal antibodies to select VAR2CSA antigen-expressing parasitized erythrocytes concluded that both *var2csa*-type genes were expressed and up-regulated following this selection [55].

The gene mapping studies shown here prove that two or more related, but not identical *var2csa*-type genes exist in many *P.falciparum* isolates. Although there is a conserved locus on chromosome 12, the positions of the other *var2csa*-type gene loci appear to vary between genomes and are currently mapped to chromosome 1, 8 and the chromosome 5–8 aggregation. In two cultures of monoclonal isolates containing multiple *var2csa*-type genes (Tz748 and Tz745 respectively), we have shown that both genes are transcribed and neither is an inert pseudo gene. Gene mapping insights into the unexpected complexities of the *var2csa*-type genes illustrates the need for genetic assays in addition to sequencing when studying gene/phenotype interactions in large multi-gene families. Chromosome mapping has recently revealed another PfEMP1/*var* gene duplication, in which the It*var*4 gene has been duplicated and transposition *via* mitotic recombination in culture, from within the chromosome 9–7 aggregation, into a site adjacent to the chromosome 12 *var2csa*-type gene [56]. Again, the chromosome 12 position of a *var2csa*-type gene remained conserved in these CS2/EB8 lineage parasites (derived from the FCR3/It4 lineage), where it functions as an expression site for transcription of full-length mRNA from both the *Itvar4* and the *var2csa*-type gene [57].

Our quantitative PCR assays indicate that the expansion of the *var2csa* sub-family is a common feature of natural populations of *P.falciparum* isolates. Around twenty percent of the isolates examined and up to a third of Tanzanian placental isolates contain two or more *var2csa*-type genes/haploid genome. The fact that a higher proportion of placental isolates have multiple *var2csa*-type genes than occur in samples from non-pregnant individuals may indicate that the multi-locus genotypes have a selective advantage in placental malaria infections. However, larger sample sizes are needed to confirm or reject the hypothesis that multiple *var2csa-*type genes are a polymorphism which confers greater antigenic variation capacity or a gene dosage and increased adhesion production advantage to the multi-locus genotype parasites in a placental malaria infection, but not in other host infections.

Where two *var2csa*-type genes exist in one genome, the sequences of each gene do not seem to be more closely related to each other than either is to any other randomly compared *var2csa*-type sequence in a different genome. In addition, neither of the *var2csa*-type sequences that have been sequenced in a multi-copy genome displays any unique sequence variation and they have the same polymorphic positions as all other *var2csa*-type sequences. These results imply that the multiple copies of *var2csa*-type genes found in some clones are not recently duplicated paralogs experiencing relaxed functional constraints.

Our analysis of the VAR2CSA DBL2X sequences found a 26 amino acid dimorphic sequence, the largest dimorphic region yet identified in a PfEMP1 gene. Structural modeling of one DBL2X variant combined with ligand binding and antibody epitope mapping studies indicate that this dimorphic sequence is surface exposed on the VAR2CSA antigen, binds the glycosaminoglycan ligand CSA and is also a binding site for IgG antibodies from women who had suffered pregnancy associated malaria [58]. This motif also seems to be conserved in the relatively distantly related *P.reichenowi var2csa*-type gene. Together, these observations argue that this dimorphic sequence and this region of the antigen have some function which has been conserved through a significant period of the evolution of the *P.falciparum* lineage of malaria parasites. The existence of strong linkage disequilibrium between dimorphic variant types seems unlikely as *var2csa*-type gene loci occur on different chromosomes and will be randomly re-assorted during the *P.falciparum* meiosis in the insect vector.

Although it has not previously recognized in PfEMP1 proteins, regions of dimorphic variation have also been reported in several other major *P.falciparum* antigens, notably the merozoite surface antigens MSP-1 [59], MSP-2 [60], MSP-3 [61], MSP-6 [62] and EBA175 [63]. Various speculative evolutionary explanations for this notable and peculiar type of polymorphism in genes encoding *P.falciparum* antigens have been proposed. These include adaptation to divergent local host populations, ancient speciation and re-unification, recurrent gene duplication and deletion and population bottlenecks (reviewed in Roy, Ferreira & Hartl [64]).

Our data are noteworthy because the *var2csa*-type genes are the only PfEMP1/*var* gene so far shown to have recognized dimorphic sequences. This is possibly related to them also being the most conserved of the *var* genes. Thus the *var2csa*-type genes have retained some evidence of dimorphic and, unlike the merozoite antigen genes so far analyzed, are occasionally duplicated. This gives a precedent, and some support for the hypothesis that dimorphism in *P.falciparum* antigens may be the result of paralogous evolution following gene duplication, followed by later loss of one copy [65].

Our data indicate that dimorphic sequences tends to disappear more rapidly from the fastest evolving genes encoding the recombinogenic [66] and hyper-variable [67] PfEMP1 antigens than from the genes encoding the highly polymorphic, but relatively more slowly evolving merozoite surface antigens. The fact that this distinct dimorphic region still distinguishes the DBL2X domain of all known VAR2CSA sequences argues that each dimorphic variant has some essential function. The VAR2CSA DBL2X dimorphic sequence is in fact an immuno-dominant region of that antigen [58]. It is interesting that this experimental observation was predicted by earlier models for the maintenance of such dimorphic antigenic polymorphisms in *P.falciparum* evolution [68]–[70].

## Supporting Information

Figure S1 Sequence alignment of full-length VAR2CSA DBL2X domains. In the P.falciparum 3D7 reference genome this domain includes amino acids 535–878 of the VAR2CSA sequence encoded by the PFL0030c gene. The multiple sequence alignment of these approximately 343 amino acids includes 37 VAR2CSA DBL2X sequences derived from Senegalese placental isolates and 18 database-derived sequences, including P.reichenowi. Color blocks mark alignment positions with >55% amino acid sequence identity. The dimorphic sequence motif (DSM) is shown between positions 50–78.
(10.42 MB TIF)
