# Supplementary figures and images for: Multiple var2csa-Type PfEMP1 Genes Located at Different Chromosomal Loci Occur in Many Plasmodium falciparum Isolates
Source: PLoS One. 2009 Aug 19;4(8):e6667. doi: 10.1371/journal.pone.0006667 (PMC2723927; doi:10.1371/journal.pone.0006667)

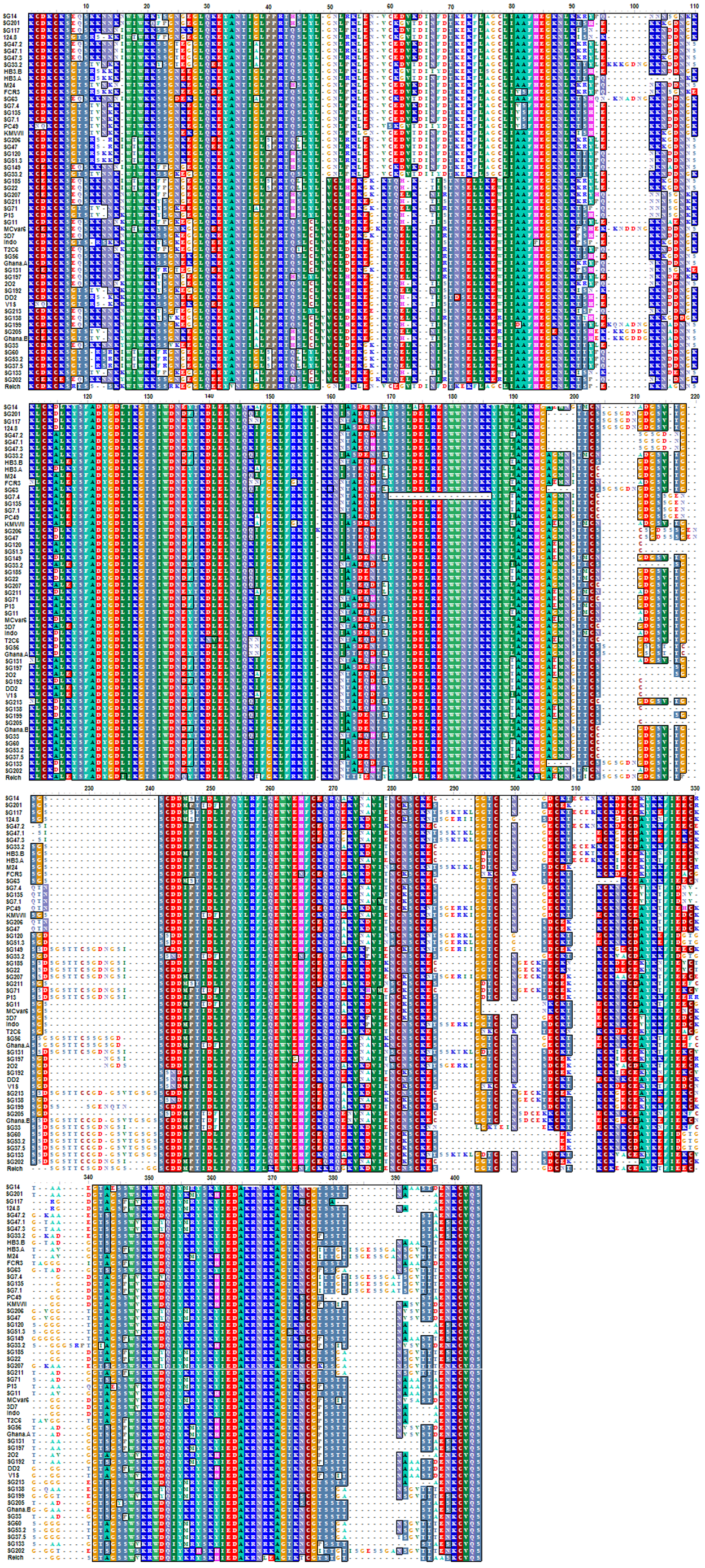

Supplement: Figure S1 — Sequence alignment of full-length VAR2CSA DBL2X domains. In the P.falciparum 3D7 reference genome this domain includes amino acids 535–878 of the VAR2CSA sequence encoded by the PFL0030c gene. The multiple sequence alignment of these approximately 343 amino acids includes 37 VAR2CSA DBL2X sequences derived from Senegalese placental isolates and 18 database-derived sequences, including P.reichenowi. Color blocks mark alignment positions with >55% amino acid sequence identity. The dimorphic sequence motif (DSM) is shown between positions 50–78. (10.42 MB TIF) [file pone.0006667.s001.tif]
